# Life beyond diagnosis: My journey of hope

**DOI:** 10.4102/sajpsychiatry.v32i0.2714

**Published:** 2026-06-19

**Authors:** Edward Nkurunungi

**Affiliations:** Department of Administration, Peer Nation, Kampala, Uganda

My name is Eddie Nkurunungi. When I was born, my mother asked the sex, and they said, ‘it is a boy’. So, she said, ‘good news’: *Nkuru* is news, *nungi* is good. So, I became Nkurunungi, the gospel.

My family comes from Southwestern Uganda, Ahamunyinya Village: a place where acacia trees are found. My dad was a headteacher but moved to Kampala to work for Save the Children, then the United Nations (UN): so, I was raised as a city kid in a block of flats, speaking many languages, Luganda, Swahili, Arabic, Nubian and our native Rukiga.

I enjoyed school, but was a bit naughty, in trouble for speaking in class, and speaking our native language vernacular, which was punishable: you are meant to speak English only at school. They would read out names of ‘vernacular speakers’ at assembly, and you would be smacked.

My mother sold used clothes from Europe and had a grocery shop. She was welcoming but had a problem with alcohol. This brought conflict with my dad, and they would fight. They separated when I was five, and we moved to Mbarara with my mother.

We came back to Kampala when I was eight, and I was popular at school and fast at making friends. When I joined secondary school, I was in rhyme with the establishment and made head prefect.

I experienced the first signs of mental illness at tertiary college while running for election. There was pressure to fundraise to grease the hands of voters, and there was witch-hunting and backstabbing from my opponents, looking for negative things to tarnish my name. The pressure strained me.

I did not feel safe. I left my roommates, moved in with my girlfriend, then my uncle, where I barricaded myself with furniture at the door. Neighbours broke the door and manhandled me to the police, where a psychiatric clinical officer (PCO) tried to talk to me but realised I was violent and sedated me.

I woke naked on a concrete floor, with no toilet or water. Attendants would bring food and close the door again. I tried to get out, but there was no window; I tried to call for help, but nobody would come. After a few days, my parents visited, and I got some fresh air. I was in uniform shorts without a shirt, saw other people in uniform and realised that I was locked up in hospital. I did not even know that place existed.

After a few days, I saw a psychiatrist. I was disoriented and could not comprehend. They forced me with medication, sedatives and antipsychotics. I asked what they were treating, but nobody would answer.

I did not see the need to stay in the hospital and helped myself out: there was no serious fence, and I just left. I walked through villages, got money for a taxi, but they said, ‘go away, go back’ because of the uniform shorts I was wearing, they were stigmatising me. I guessed my college was in the west, so I followed the sunset. At college, friends saw me in a sorry state, disoriented and out of focus, in uniform shorts and with wounds all over my body: they were supportive and kind and got me to my parents.

I received treatment from the PCO that first saw me. Later on, I understood that they were treating bipolar disorder but at the time, they just said ‘you’re *mulalu*’ which means ‘mad’. The treatment was *carbamazepine* that would make me shake like a leaf, dribble and doze off. Eventually, the fear, paranoia, restlessness and overtalking receded, and I returned to college to complete my studies.

In 1999, my mother died, which devastated me and left a vacuum in my life. I decided to join my siblings in London and stayed with my sister in her one-bedroom flat.

In the United Kingdom (UK), I faced a cultural clash: life was fast, and I had to work hard to make ends meet: cleaning toilets from 06:00 then working in a restaurant until 23:00. Within 2 weeks, I had broken down mentally.

I was not sleeping and barricaded myself in the house, paranoid that police were looking for me. My sister called the police, who broke the door but let me go, so I started to look for the Ugandan High Commission to help me get home.

I walked the whole night, but everything was too fast. I was racing with cars, stripped naked, jumped over a fence, and I was brought down by the police. They asked my name: I did not know whether to tell them a pseudonym or my actual name. They took me to a psychiatric hospital. They gave me carbamazepine and told me it was bipolar. I was discharged, but they did not give me a follow-up plan, because they thought that I was going back to Uganda.

I found a new job under a pseudonym, but had a constant fear that the police would find out I was working illegally. This increased to paranoia: I thought the police were coming and assaulted my sister. I was confused, my mind was too much, and I thought of jumping off the balcony. The police arrested me and then took me to the hospital.

I was taken to court and was found ‘not guilty’ by virtue of insanity: they recommended treatment in a secure setting without limit of time, under home office restrictions. I then spent the next 7 years in a hospital in incarceration.

My diagnosis changed to paranoid schizophrenia. I had never heard of that diagnosis and even now, I do not resonate with schizophrenia: although I experience paranoia, I do not experience hallucinations.

I thought they were racist to give me this diagnosis, because the index offence was violent. I did not understand the intensity of the diagnosis; it was just some verbose words. But when you read about schizophrenia, the prognosis is poor, the chances of recovery are not there. It would mean I would never function on my own; never be able to sustain a job, have a family or raise children. I would not be responsible, just some hopeless person who cannot achieve anything.

At my first tribunal, my diagnosis changed to schizoaffective disorder because of my history of bipolar. These were words that I was not familiar with, but I learned that the prognosis of schizoaffective disorder was more positive: people can recover. My treatment was reduced to a monthly injection.

There was a helpful psychotherapist whom I saw for 5 years, who helped me understand the genesis of my mental disorder in my upbringing as a child.

Occupational therapy was the most helpful to get routine, shopping and cooking, interacting with peers, undertaking IT training, getting paid roles, cleaning the ward and working in the canteen.

As Foundation Trusts were launched, there was a requirement for service user representation, and I would meet with clinicians, who listened to our experiences of institutional settings, the care we needed, the life we wanted: that is how I started my journey with the service user movement.

At my last tribunal, my diagnosis was changed back to paranoid schizophrenia-in-remission because I had no mood swings or extremes of energy during my 7 years in hospital. I then returned to Uganda in August 2007.

When I left Uganda, I had a culture shock in London, but when I returned, I got an aftershock because so much had changed. My friends had moved on, were now big guys, in government, with families. I could not relate to them and saw myself as an underdog, as someone who had gone to the UK and wasted his time in a secure setting, coming back with nothing to show.

I felt out of place, but in the service user movement, I found people who believed in me and accepted me for who I am. I felt comfortable and, with time, started opening up. In London, I was told, ‘don’t tell people what happened to you, don’t share your index offence, just keep it to yourself’. But now I have found peers who were outspoken and proud of their identity as service users with lived experience. Being part of Heartsounds helped me spread my wings.

Joining the mental health service user movement has brought meaning and purpose. I coordinated the Butabika Recovery College and founded Peer Nation, a non-governmental organisation (NGO) of which I am the Chief Executive Officer (CEO). I have coordinated Peer Support Workers training and working all over Uganda, in the community and in refugee settlements; coordinated the Brain Gain and Using Peer Support in Developing Empowering Mental Health Services (UPSIDES) projects, secured funding from Department For International Development (UK Aid) (DFID) and/or Tropical Health and Education Trust (THET), the Storm Fund, Open Society Initiative for East Africa (OSIEA) and the Disability Rights Fund. I have met ministers, lobbied for the Mental Health Act (MHA) reform on radio and television, and travelled to Tanzania, Rwanda and South Africa to talk about service user involvement. This is the legacy I would wish to leave behind.

There is more to a human being than a diagnosis. A diagnosis can be transient, a working hypothesis that could be proved right or wrong: it should never be cast in stone. It is the people and the stories behind their diagnosis and labels that matter the most.

